# Applications of multiplexed CRISPR–Cas for genome engineering

**DOI:** 10.1038/s12276-025-01500-6

**Published:** 2025-07-31

**Authors:** Himchan Cheng, Euihwan Jeong, Seung Woo Cho

**Affiliations:** 1 Department of Biomedical Engineering, College of Information and Biotechnology, Ulsan National Institute of Science and Technology, Ulsan, Republic of Korea; 2 CasCure Therapeutics, Ulsan, Republic of Korea; 3 Center for Genomic Integrity, Institute for Basic Science, Ulsan, Republic of Korea

**Keywords:** Gene targeting, Epigenomics

## Abstract

The CRISPR–Cas system has become a worldwide genome editing tool for various organisms. Its precision and efficiency have facilitated basic research, drug discovery and therapeutic interventions. In contrast to other genome editing agents, CRISPR–Cas is modulated by a short guide RNA. Due to its simplicity, CRISPR–Cas is recognized as the best candidate for multiplexed genome editing. With simultaneous targeting, efficient knockout of genes with large deletions is possible. In addition, CRISPR–Cas can induce complex structural variations, such as inversions, translocations and duplications. Moreover, by utilizing engineered CRISPR–Cas proteins specialized for direct repression or activation of gene expression, one can perform multiplexed epigenetic editing. Lastly, multiplexed targeting enables killing of specific types of cells by accumulating stress mediated by simultaneous DNA damages. Here we discuss how CRISPR-based editing technologies for multiple targets are applied in recent studies.

## CRISPR–Cas advances multiplexed gene editing techniques

Gene editing has been an important scientific focus for decades, with CRISPR–Cas9 emerging as a groundbreaking tool. This study explores the ability of CRISPR–Cas9 to edit multiple genes simultaneously, which is crucial for understanding complex genetic interactions. Researchers demonstrated that CRISPR–Cas9 could efficiently edit multiple sites in human cells. This method is particularly useful for creating large deletions or rearrangements in DNA, which can mimic genetic diseases or help develop new treatments. Furthermore, use of CRISPR activation and CRISPR interference enables simultaneous activation and repression of genes. The study concludes that the ability of CRISPR–Cas9 to target multiple genes at once opens new possibilities for research and therapy, especially in cancer treatment. For instance, targeting multiple cancer-associated sites with CRISPR–Cas9 selectively kills cancer cells. Together, this Review presents various applications of multiplexed genome editing.

This summary was initially drafted using artificial intelligence, then revised and fact-checked by the author.

## Introduction

**Fig. 1:**
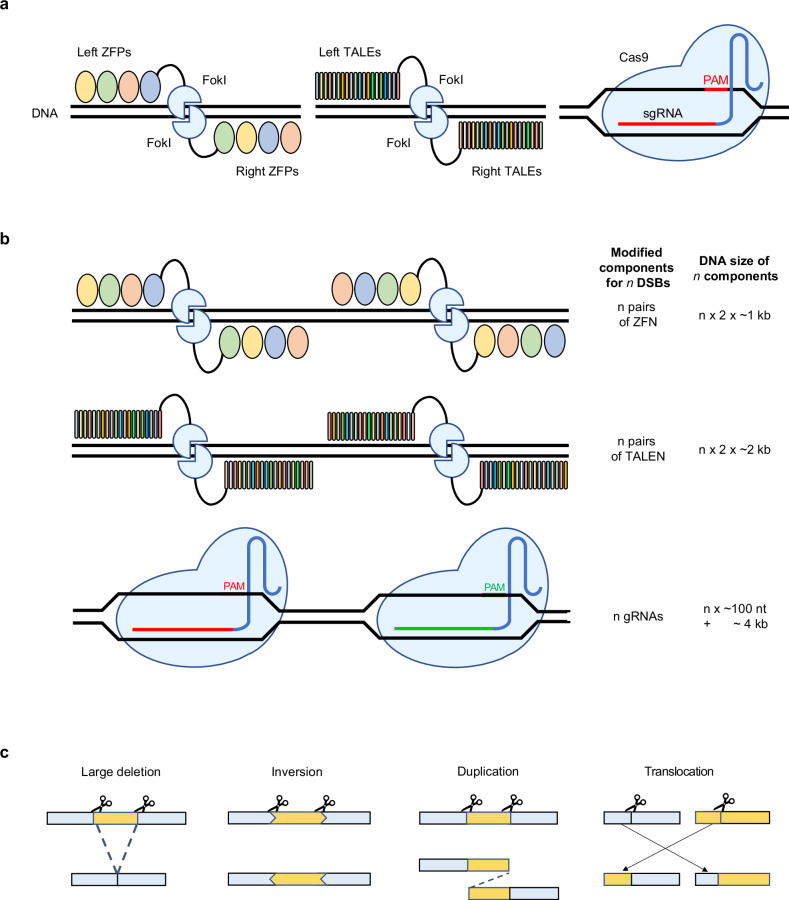
Multiplexed genome editing technology is capable of generating complex SVs. **a** An illustration of different types of genome editing technology: ZFN (left), TALEN (middle) and CRISPR–Cas9 (right). **b** To edit different target site, components of genome editor recognizing targeting region should be modified. The modifications required depend on the type of genome editor. CRISPR–Cas9 is more efficient for engineering than ZFN or TALEN because it is guided to target DNA by short gRNA. **c** Two concurrent DSBs can induce various SVs. Four common applications are large deletions, where DNA between two DSBs are deleted, inversions, where deleted fragment is inversely repaired, duplications, where repair occurs with duplicated fragment between breaks, and translocations, where broken chromosomes are attached to each other.

Decades have passed since the first trials made for DNA modification, yet gene editing has not declined from its zenith. The discovery that DNA double-strand breaks (DSBs) enhance gene targeting—which otherwise has a low success rate in higher eukaryotic cells (typically 10^−7^ to 10^−6^)—highlighted the need to develop effective methods for introducing site-specific DSBs into cells^1^. Scientists sought the solution from biological sources: enzymes. Appliance of meganuclease I-SceI^2,3^, zinc finger nuclease (ZFN)^4–8^ and transcription-activator-like effector nuclease (TALEN)^9^ all revealed promising results, yet were not so convenient for broader usage as a comprehensive toolbox for genome research (Fig. 1a).

The impact of the first demonstration of gene editing using CRISPR–Cas9 was thus phenomenal. Unlike its predecessors, CRISPR–Cas9 requires less effort for target design. That is, its amino acids need not be engineered to bind at different target sites. Rather, one can modify any genomic region by simply swapping guide RNAs (gRNAs) complementary to the target site as long as the protospacer adjacent motif (PAM) is present. The total length of Cas9 gRNA is approximately 100 nt, with only 20 nt responsible for target specification. Meanwhile, switching the target site for ZFNs and TALENs involves designing a long array of DNA-binding motifs (Fig. 1a,b). Moreover, many studies have consistently reported that the ability of CRISPR–Cas9 to promote site-specific mutations persists across various mammalian cells, including human cells^10–12^, as well as in plants^13–15^. Collectively, these advances marked the beginning of the new era of genome engineering.

Soon after CRISPR–Cas9 was recognized as an agent for DNA modification, the science community was flooded with implementation of CRISPR–Cas for numerous purposes. Disruption of genes was the first widely applied technique using the CRISPR system^11^. The main goal is to trigger error-prone nonhomologous end-joining (NHEJ) by DSBs through endonuclease activity. Repaired DNA containing errors, mostly insertions or deletions (indels), may suppress or alter the function of targeted genomic element. Furthermore, researchers who understood the outstanding modularity of CRISPR–Cas integrated domains allowing the manipulation of gene expression^16–20^, base conversions^21–23^ or DNA replacement^24^.

Successful use of CRISPR cannot be taken for granted without careful study design and strategic planning. One of the most promising approaches for gene perturbation is dual target editing. This technique relies on two simultaneous DNA breaks to cause a large deletion of the genomic region, which in turn disrupts proper functioning of the targeted gene and noncoding elements^25–30^. In addition, dual target editing may lead to complex structural variants (SVs) such as inversions^31–33^, duplications^31,34^, and translocations^35–37^ (Fig. 1c). Editing more than two sites simultaneously is also applicable with CRISPR–Cas. By delivering multiple gRNAs into host cells, one can knock out^25,26,38–41^ or regulate^18,39,42–44^ multiple genes at once.

One important concern with those methods is the cellular damage caused by simultaneous DSBs. Considering possible cytotoxicity, allowing multiple gene edits at once using nucleases may be considered premature and impractical by those seeking medical usage of simultaneous CRISPR activities. However, the situation is not necessarily hopeless. A recent report by our group suggests that numerous targeted DSBs specific to cancer cells cause cell death in cancer cells but not in normal cells^45^. The results of the study are meaningful because they offer a new possibility of CRISPR-mediated cancer therapy.

This Review aims to cover the details of well-established applications utilizing CRISPR–Cas technology with multiple gRNAs. These include applications of simultaneous editing, examples of structural variations mediated by CRISPR double-targeting, multiplexed epigenome editing and, finally, studies on multiple DNA damages. This Review also provides agricultural usage of CRISPR-based multiplex editing and highlights the flexibility of the CRISPR–Cas platform in producing targeted DSBs with high precision and efficiency.

## Simultaneous gene knockout

The first published work presenting a multiplex CRISPR–Cas system was reported by Cong et al. from the Zhang group. They optimized Cas9 and gRNA to be efficiently expressed in human cell lines and observed various alleles made by nuclease activity. Testing multiple gRNAs targeting the *EMX1* locus, they concluded that, although efficiency differs from site to site, Cas9 is widely applicable in human cells. Based on its generalizability, the authors hypothesized it would also be possible to activate Cas9 at two different targets together. Two studies were designed to prove Cas9’s potential for multiplexing: targeting two different genes (*EMX1* and *PVALB*) and targeting two sites on the same gene (*EMX1*). In both cases, Cas9 effectively modified both sites. Importantly, cleavage at two *EMX1* sites created a large deletion causing a frameshift in the *EMX1* codon, suggesting that multiplex genome editing using CRISPR-Cas is capable of targeted gene knockouts^25^.

The ability to induce sufficient target-specific mutations is a crucial factor for studies involving gene knockouts. As the function of coding genes and noncoding elements remain largely unknown, there is great interest in solving the enigma of the human genome. The common practice to do so is genome-wide screening. The key to such a screen is loss of function; examining the effects on cell viability after disturbance of certain genes may provide insights into the underlying features of those genes^46–48^. CRISPR–Cas9 is well suited for this purpose because of its simplicity in designing target libraries and its high efficiency in perturbation.

In most cases, a single knockout may be sufficient. However, this approach is limited in some respects. First, single-knockout systems cannot directly reflect interactions between genes. Second, a single DSB does not guarantee loss of function for noncoding elements. Therefore, combinatorial knockout to introduce large, paired deletions would be a better strategy in such cases.

The CRISPR-based double-knockout (CDKO) library designed by the Bassik group is one of the earliest works attempting to implement a dual CRISPR system in a genome-wide functional screen. Notable features of CDKO arise from the carefully designed structure of its lentiviral vector. The main concern with placing two or more gRNAs with repeated use of identical promoters in a single vector DNA is the potential for recombination due to homologous sequences between promoters. To address this, creators of the CDKO library used human U6 and mouse U6 promoters to express each of the two gRNAs. Importantly, the library is also constructed in a way that is convenient to clone a huge set of randomized pairs and compatible with popular Illumina short-read sequencing platforms. The authors demonstrated the utility of their library by identifying synthetic lethal targets of drugs in K562 from 490,000 gRNA pairs^38^. It is also worth noting that multiplexed CRISPR screening is shown to be adaptable with single-cell RNA sequencing^39,41,48^. Adamson et al. made a CRISPR screening library containing three gRNAs and linked them with single-cell RNA sequencing results to reveal mammalian unfolded protein responses^39^.

Similarly, Zhu et al. generated the first paired gRNA lentiviral CRISPR library to delete and examine phenotypes of long noncoding RNAs (lncRNAs) in a high-throughput manner. Among the 700 human lncRNAs they tested, 51 lncRNAs were identified as either positive or negative regulators for liver cancer proliferation^40^. More recently, Li et al. developed a dual gRNA library targeting noncoding elements other than lncRNAs: enhancers and ultraconserved elements. By doing so, a regulatory element that was previously unknown was identified in the K562 cell line^41^.

In cancer research, the more genes that are targeted, the greater the insight that can be gained. With the increasing popularity of CRISPR-mediated technologies, the demand for strategies to effectively edit two or more targets simultaneously has also increased. As with double-targeting CRISPR libraries, construction of three or more targeting systems requires a unique strategy. Among the most popular is the Golden Gate assembly. With this cloning method, gRNA-containing DNA fragments digested by type IIS restriction enzymes fit together in specific orientations^49,50^. Sakuma et al. directly applied Golden Gate assembly and constructed a single CRISPR–Cas9 cassette with seven gRNAs^49^. To advance the approach, Zuckermann et al. developed a ‘PCR-on-ligation’ step, allowing the modular assembly of multiple gRNAs, and successfully demonstrated 10-plex gene editing in the HEK293T cell line using their method^50^. A notable result from their experiments is that multiplexed targets were modified at levels similar to those of individual targeting^49,50^.

## Simultaneous genome editing using nickases

Ran et al. further improved the methodology of CRISPR editing. These researchers realized that fully active Cas9 often creates off-target mutations. They focused on two different nuclease domains, HNH and RuvC, of Cas9. Reasonably assuming that inhibiting the catalytic residue of either domain would result in targeted DNA nicks instead of full DSBs, and therefore probably trigger high-fidelity base excision repair rather than NHEJ, Ran et al. tested and compared Cas9 nickases with Cas9 nuclease in terms of target specificity. In their experiments, two Cas9 nickases were programmed to target opposite DNA strands to mediate DSBs^26^. The group successfully demonstrated that two simultaneous nicks can efficiently cause mutations on-target with reduced off-target activities.

According to studies, nicks can also be corrected by another repair mechanism called homologous recombination (HR)^26^. In this case, homologous chromosomes can be used as template DNA to fix the nick. Motivated by this property, Tomita et al. attempted to restore heterozygous mutations with Cas9 nickase. Although a single nick on a target gene corrected some mutations, the effect was negligible. Tomita et al. then increased the number of nicks and targeted both donor and recipient alleles. Surprisingly, multiple nicking achieved tenfold higher efficiency in enhancing gene correction. Multiple nicks rarely generated short indels at both on-target and off-target sites, indicating the strategy to be a safe editing approach. Moreover, HR induced by multiple nicks proved to be capable of repairing nonsubstitutions such as small indels and large deletions^51^.

## CRISPR-induced structural variation

Loss of genetic elements may elicit new inference about the previously unknown biological system. Investigating already-known phenomena may also produce rich information for our understandings. According to reports, the expected similarity between DNA sequences of two different people is about 99.9% (ref. ^52^). Sometimes, these small differences in DNA may greatly contribute to one’s susceptibility to diseases. From the efforts to identify causal genetic variations, SVs turn out to be major factors in enhancing some adverse phenotypes^53^ (Fig. 1c).

Nevertheless, characterizing the full impact of cancerous SVs requires appropriate models. To imitate SVs under various circumstances, researchers took advantage of customizable endonucleases^31–36,27,28^. Brunet et al. reported the first success of ZFN-derived translocations^35^. Following this achievement, many translocations and other SVs relevant to different types of cancer were imitated with ZFNs and TALENs^31,32,27^. It is not surprising that CRISPR–Cas9 replaced these endonucleases and became the most popular tool for producing artificial SVs. By using dual gRNAs, Choi and Meyerson used CRISPR–Cas9 to induce several pathogenic rearrangements in lung cancer, including *CD74*–*ROS1*, *EML4*–*ALK* and *KIF5B*–*RET* fusions^36^. Torres et al. programmed abnormal *EWSR1*–*FLI1* and *RUNX1*–*ETO* fusions that are commonly found in Ewing’s sarcoma and acute myeloid leukemia, respectively^37^.

As much as it is capable of creating complex mutations, CRISPR–Cas9 is also successful in reverting existing SVs. In 2015, Park et al. applied CRISPR–Cas9 to correct chromosomal inversion of the *F8* gene in induced pluripotent stem (iPS) cells derived from patients with hemophilia A. Those reprogrammed iPS cells were lacking any significant off-target edits and successfully expressed normal *F8*^33^. Young et al. deleted up to 725 kb of DNA between exon 45 and exon 55 of the *DMD* gene, where 60% of mutations corresponding to Duchenne’s muscular dystrophy occur, in human iPS cells^28^. Li et al. generated inversions and duplications of the protocadherin clusters in mice^34^. These studies indicate the wide applicability of the dual gRNA system in the medical field.

In many cases, however, observing systematic changes followed by a gene knockout is a more straightforward approach for model studies than generating complex variants. As large deletions are more reliable than small indels in terms of complete gene knockout, CRISPR-mediated large deletions have been the preferred form of artificial SV^29,30^. Although the method itself seems simple enough, isolating cells with completely removed genes requires thorough cell selection for biallelic mutations, which remains a challenge. To help streamline this laborious process, CRISPR-del provides a pipeline to delete DNA spanning hundreds of kilobases and subsequently examine the genotypes resulting from the deletion^29^.

Besides their usefulness, DSB-based large deletion techniques have problematic downsides. In particular, the NHEJ pathway carries unintended and unpredictable indels or complex variations at either or both DSB sites. To resolve the issue, gene editors that yield more precise variations are necessary^24^. Choi et al. identified Cas9 prime editor (PE) as a suitable candidate^30^. A key feature of the PE is inherited from Cas9 nickase; it produces a targeted nick, circumventing error-prone DNA repair. In addition to nicking DNA, the PE uses the special form of gRNA called pegRNA containing a template sequence and reverse transcriptase to precisely integrate a single-stranded DNA with desired sequence ‘flapping’ at the nicked site^24^. While repairing the nick, this flap DNA is incorporated into the target DNA. By directing two PEs to target opposite strands, facing each other, Choi et al. precisely deleted up to 10 kb of DNA. Another benefit of PE-based large deletion is that it can replace the deleted sequence with favorable ones, allowing more flexible usage^30^.

## Multiplexed epigenome editing

Up to this point, applications of CRISPR–Cas9 have relied on DNA breaks. Although CRISPR–Cas9 is inherently an endonuclease, it is not necessarily bound by its original function. Regarding its specificity, Qi et al. developed catalytically inactive, or dead, Cas9 (dCas9) for modular protein binding at sites of interest. They engineered dCas9 to block the initiation and elongation of transcription, thus downregulating the expression of genes of interest^13^. Further studies elaborated this platform by fusing effector proteins with dCas9. Based on the type of linked effector, dCas9 may either repress (CRISPR interference, CRISPRi)^16,17,43^ or facilitate (CRISPR activation, CRISPRa)^18–20,42,43^ gene expression.

As expected, multiplexing these regulations with CRISPRi and CRISPRa systems is also possible. In fact, multiplex expression control is desirable in cases where complex genetic interactions are involved. Two main approaches can be taken: using separate gRNAs to control multiple genes simultaneously^18,44,54^ or directing multiple gRNAs to the same gene for enhanced editing^42,43^.

Konermann et al. used the first approach with their unique CRISPRa system, the synergistic activation mediator (SAM). They tested the simultaneous activation of ten different genes using SAM and observed activation efficiencies comparable to those of single reactions^18^.

However, delivering multiple gRNAs separately becomes inefficient as the number of gRNAs increases. Fortunately, another CRISPR system, CRISPR–Cas12a, lessens the burden. The major difference between Cas9 and Cas12a is that Cas12a can self-process a series of gRNAs in an array into individual gRNAs. This feature enables a single promoter to express various Cas12a gRNAs at once^54–58^. The paper published by *Nature Methods* introduces an original platform called single-transcript Cas12a (SiT-Cas12a). Here, Cas12a and its gRNA array are expressed by a single EF1a promoter. SiT-dCas12a-KRAB was shown to efficiently knock down four different genes simultaneously. Moreover, SiT-dCas12a-p65-HSF1 activator enhanced ten distinct genes by 10- to 1000-fold^54^.

**Fig. 2:**
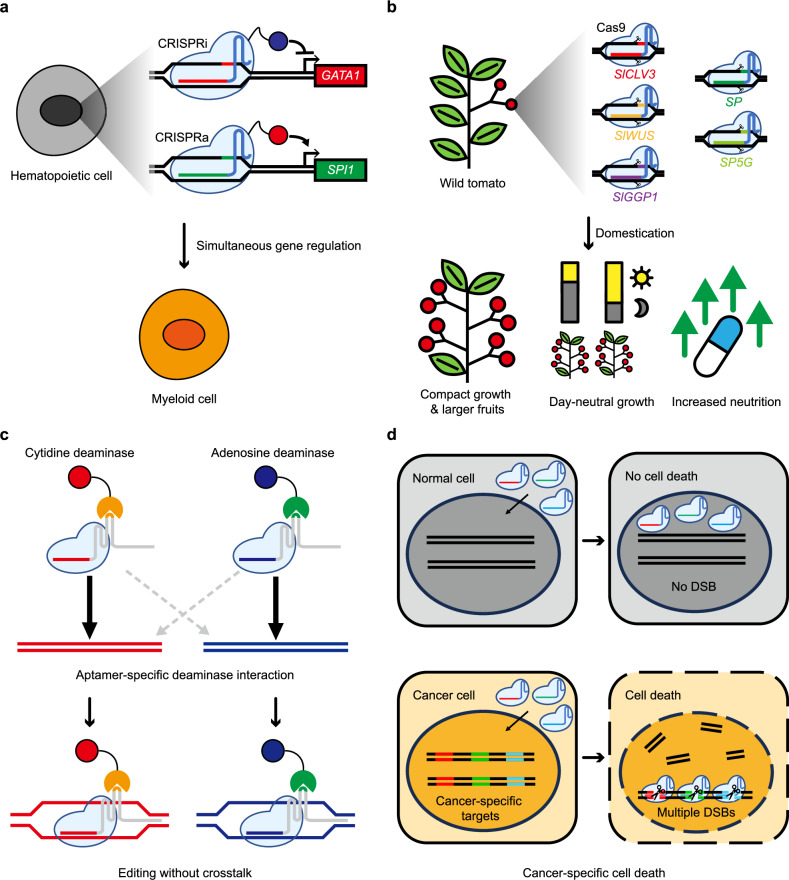
Multiplexed editing is widely applied to biological science. **a** CRISPRi and CRISPRa can be used at the same time for sophisticated gene regulation. In the illustration, repressed *GATA1* and stimulated *SPI1* are shown to cause the differentiation of a hematopoietic cell. **b** Multiple traits of crops can be engineered simultaneously by CRISPR–Cas. The figure illustrates beneficial changes made for wild tomatoes by multiplexed editing. **c** With the help of gRNAs with deaminase-specific aptamers, the MOBE system enables simultaneous base editing of different types without crosstalk. **d** DSBs cause cellular stress. By targeting multiple cancer-specific mutations at once, one can induce cancer-specific cell death.

The combination of both CRISPRa and CRISPRi is also helpful for understanding complex genetic networks. With bidirectional editing method, called CRISPRai, researchers demonstrated simultaneous repression of *GATA1* and activation of *SPI1*. By doing so, they enhanced the modulation of cell lineage signatures (Fig. 2a). The regulatory landscape of *IL2* was also explored, identifying mechanisms of enhancer-mediated gene regulation in various T cell contexts^44^. These findings suggest that bidirectional co-regulation may provide new insights into functional interactions between genetic elements.

Some researchers pointed out that early CRISPRi and CRISPRa systems do not alter chromatin directly. This concern is supported by recent advances in sequencing technology that revealed the importance of epigenetic features in affecting gene expression^42,43^. To compensate for the limitation, further developments to CRISPRi and CRISPRa were made. Integrating the catalytic core of human acetyltransferase p300 into dCas9 led to acetylation of H3K27 and, thus, activated the expression of nearby genes^42^. Heterochromatin protein 1 as a CRISPRi effector forms a complex with several methyltransferases and causes trimethylation in H3K9, suppressing nearby gene expression^43^. In these cases, predicting a single ‘hot spot’ enhancer region to silence or activate is challenging. To accommodate efficient engineering, they targeted multiple Cas9-targetable enhancer regions. No matter the platform, all experiments with pooled gRNAs were successful^42,43^.

## Plant editing

Just as the academic community is keen on uncovering novel insights into human biology, it is equally invested in advancing our understanding of plants. Experts have consistently emphasized the importance of innovative solutions in agriculture to meet future demand for staple crops, which are major sources of food^13^. To address this challenge, technology that can stabilize crop yields should be developed. Fortunately, CRISPR–Cas gene editing has proven to be a powerful tool for engineering plant traits^13–15,59^.

Currently, most manipulations in crops are done to increase yield and quality and to protect them from disease. Clearly, these need not be done separately when guided by CRISPR editing. Simultaneous editing of six genes in wild tomato can introduce multiple advantageous traits, including day neutrality, enlarged fruit size and increased vitamin C levels^14^ (Fig. 2b). Such editing enables de novo domestication of the species without decreased fitness and genetic diversity.

However, conventional multiplexed editing using one type of CRISPR system with multiple gRNAs is not enough for complex genomic changes. Various Cas9 orthologs are applicable for DNA editing, and interestingly, each Cas9 endonuclease recognizes different PAM and gRNA sequences^60,61^. Considering this characteristic, some researchers simultaneously deliver different orthologs into cells to target an expanded scope of genomic regions. For example, Hua et al. utilized variants of SpCas9 and SaCas9. They incorporated these variants into new base editors to modify the rice genome. Although the base editing efficiency varied across different target sites, this study successfully demonstrated the potential for simultaneous editing in rice, which is a promising development for crop engineering^15^.

## Orthogonal editing

No matter the subject—humans, plants or other species—genetic variants play an important role in functional genomics. Despite the discovery of many genetic variants, most of the known variants are rare or specific to certain populations. Even with the help of genome-wide association studies, accurately identifying rare variants and, thus, establishing prediction models are challenging, made even more difficult when multiple variants coexist. Making connections between these variants is not an easy task^62^.

As explored in earlier topics, various types of gene editor can be used for various purposes. Comprehensive gene editing with a combination of different editing utilities may enhance research on understanding the complex nature of genetic variants. This particular type of DNA editing is now referred to as orthogonal editing, and many innovative systems have been made^59,63^.

Simultaneous and Wide Editing Induced by a Single System (SWISS) substantially improves orthogonal editing in plants. SWISS uses RNA aptamers that recruit specific binding proteins. These proteins are fused with cytidine deaminase and adenosine deaminase, enabling the simultaneous execution of cytosine base editing and adenine base editing, respectively. By utilizing different Cas9 orthologs, SWISS can perform multiplexed orthogonal base editing and even induce indels^59^. The development of SWISS greatly improved the potential of synthetic manipulation of plant genome.

Multiplexed orthogonal base editor (MOBE) is also an advanced modular orthogonal editing system. Instead of using different CRISPR variants to avoid crosstalk, MOBE utilizes an aptamer-based system. Here, the modified RNA loop in the gRNA acts as an aptamer, recruiting coat protein specific to either cytidine deaminase or adenosine deaminase. Because base editing occurs only in the presence of the aptamer–coat protein complex, simultaneous base editing with minimal interference is possible (Fig. 2c). MOBE has been tested in various cell lines, including HEK293T and SH-SY5Y, proving its versatility and effectiveness in different biological contexts^63^.

## Cas9-mediated multiple DSBs

Multiplex CRISPR reaction does not always require multiple gRNAs. In the human genome, highly repetitive regions exist and targeting these loci with CRISPR–Cas results in simultaneous editing. For researchers demanding target specificity in their studies, these multitarget sites may have little value and even be problematic. Zou and his colleagues, however, sensed the potential significance of multitargets^64^.

DNA DSBs lead to cellular damage when induced. A single DSB is often enough to kill many bacteria and yeasts^65,66^. Higher eukaryotic cells, such as human cells, can tolerate higher numbers of DSBs owing to their more sophisticated DNA damage response (DDR) pathways. Even after having done much research, biologists are still debating the details underlying these processes. To characterize aspects of the DDR better, Zou et al. utilized CRISPR–Cas9 and multitarget gRNA (mgRNA). They gathered mgRNA targets from short interspersed nuclear elements. To assess the consequences of Cas9 editing, the researchers performed chromatin immunoprecipitation with sequencing (ChIP–seq), targeting MRE11, a key protein involved in the early DDR pathway. Closely analyzing the sequencing results from MRE11 ChIP–seq, Zou et al. found that epigenetic features constitute Cas9 cleavage efficiency and that Cas9-induced DSBs affect nearby chromatin accessibility. Based on their data and implications made by them, they came up with a new model for DDR after Cas9 cleavage; MRE11 binds to the DSB site and initiates repair, and chromatins near the site open up to become more accessible for other repair factors until the repair is complete^64^.

It is also possible to utilize the DSBs caused by CRISPR–Cas9 in therapeutical applications. Zuo et al. and Celli et al. shared interest in chromosome elimination using targeted DSB. Aneuploidy, or abnormality in the number of chromosomes, is an indicator of diseases, including Turner syndrome (missing one X chromosome)^67^, Down syndrome (one extra chromosome 21)^68,69^ and Edwards syndrome (one extra chromosome 18)^69^. Although scientists recognized the emerging importance of establishment of aneuploidy models and appropriate treatments for related disorders, traditional approaches relied on impractical techniques utilizing genetic recombination^70^ and drug-targetable transgenes^71^. By targeting multiple chromosome-specific repeat sites with CRISPR–Cas9, Zuo et al. and Celli et al. demonstrated complete loss of human chromosomes^72,73^.

In addition, Kwon et al. hypothesized that inducing frequent DSBs to cancer cells would be an effective cancer treatment. To prevent critical damage to normal cells, they performed whole-genome sequencing to identify cancer-specific genomic variations to search cancer-specific Cas9 targets. To prove the concept, Kwon et al. tested 2–20,000 multitargets. Because 20-or-more simultaneous damage consistently appeared to be cytotoxic in HCT116, MDA-MB-231 and K562 cancer cell lines, they designed about 20 targets specific to each cell line. As expected, cancer-specific multitargets were effective only against the corresponding cancer cell line and were benign to other cells (Fig. 2d). This method was standardized for potential therapeutic application and designated as the cancer-specific insertions and deletions attacker (CINDELA)^45^.

## Discussion

The CRISPR–Cas system plays an important role in many, if not all, biological sciences, with a large proportion of research focused on its applications in gene therapy. Indeed, approval of CRISPR–Cas9 for the treatment of sickle cell disease by the US Food and Drug Administration proves that gene editing is not merely a research experiment; it is tangible reality^74–76^. It is likely that more improved and sophisticated utilization will be introduced in the near future. Therefore, a great portion of the upcoming CRISPR research will probably be based on multiplexing.

The advantage of multiplex CRISPR platforms is apparent. Because the genetic network of higher eukaryotes is too complex to reveal new understandings, simultaneous manipulation has proven to be an effective strategy. We showed that targeting only two genomic regions is sufficient to reveal many insights into the function of coding or noncoding elements. Furthermore, CRISPR enables precise modeling of complex cancerous genomic mutations as well as their reversal.

By increasing the number of targets, the potential of multiplex editing extends even further. Beneficial characteristics are shown to be introduced by multiple modifications. Although it is seldom used at the moment, CINDELA may be further optimized for next-generation cancer treatment. Together, these experiments offer valuable insights and guidance for future CRISPR studies.

Still, there is room left for further advancement of the CRISPR-based application. One major challenge that needs to be solved is the development of a new delivery method. Current CRISPR therapies cost US$900,000–2,100,000 per patient, limiting their access^74,77^. The fact that most in vivo delivery of CRISPR is done by adeno-associated virus (AAV) is concerning. Although AAV is expected to have a low risk of viral DNA integration into the host genome, reports suggest that AAV is not entirely free from this threat^78,79^ Moreover, the limited packaging capacity of AAV makes AAV less attractive, especially when performing multiplexed editing^80^. Higher specificity is also key to the successful deployment of CRISPR. An ideal gene editor would exhibiting little to no unwarranted modification. In the case of multiplex editing, the risk coming from low specificity is higher because of possible side effects from accumulated off-target mutations. Many natural orthologs or laboratory-made variants of Cas proteins have been tested, but improved specificity often comes at the cost of reduced editing efficiency^60,61^.

Improvement in CRISPR delivery method would ease the economic and technical burden of CRISPR therapy^81^. Lipid nanoparticles are synthetic nonviral vehicles approved for use in human tissues by the US Food and Drug Administration^82–84^. Lipid nanoparticles are capable of delivering Cas9 mRNA and gRNA-assembled Cas9 ribonucleoproteins with better capacity and lower safety risk compared with viral delivery^85–87^. Off-target issues with CRISPR may be resolved by artificial intelligence (AI) models. Generated by a large language model, OpenCRISPR-1 is the first Cas9 protein designed by AI, and it appears to be both highly effective and specific in DNA editing^88^. As the field of AI-based protein design is rapidly growing^89–91^, more sophisticated genome editors are likely to be developed in the near future. These ongoing technological achievements suggest that CRISPR innovation is far from over.
